# Hypercoagulability in Cushing’s syndrome: incidence, pathogenesis and need for thromboprophylaxis protocols

**DOI:** 10.1007/s11102-022-01261-9

**Published:** 2022-07-26

**Authors:** Richard A. Feelders, Lynnette K. Nieman

**Affiliations:** 1 Department of Internal Medicine, Division of Endocrinology, Erasmus MC, Dr. Molewaterplein 40, 3015 GD Rotterdam, Netherlands; 2 National Institute of Diabetes and Digestive and Kidney Diseases (NIDDK), National Institutes of Health (NIH), Bethesda, MD USA

**Keywords:** Cushing’s syndrome, Venous thromboembolism, Coagulation, Fibrinolysis, Thromboprophylaxis

## Abstract

Cushing's syndrome (CS) is associated with a hypercoagulable state resulting in an increased risk on venous thromboembolism (VTE). In patients with untreated active CS VTE incidence is up to 18-fold higher compared to the general population, whereas after pituitary and adrenal surgery a postoperative VTE risk between 2.6 and 5.6% has been reported. Interestingly, after surgery the VTE risk is not only increased in the first week but also during several months postoperatively. The hypercoagulable state in CS is thought to be caused, at least in part, by an imbalance between activity of pro- and anticoagulant pathways. However, changes in activated partial thromboplastin time and plasma concentrations of pro-and anticoagulant factors are not observed in every CS patient. Only retrospective studies have shown that thromboprophylaxis lowers VTE risk in CS. Future prospective studies should asses the optimal timing, duration and type of thromboprophylaxis in CS to improve VTE-related morbidity and mortality.

## Introduction

Cushing’s syndrome (CS) is a multisystem disorder that is accompanied by a high risk of cardiovascular morbidity and mortality [1]. Less well known is that CS, irrespective of the cause, is associated with a hypercoagulable state predisposing these patients to venous thromboembolism (VTE) that may contribute to the cardiovascular morbidity as well [2]. This hypercoagulable state may seem discrepant with the increased bruising of CS and might be considered as a ‘CS coagulation paradox’. However, the increased bruising of CS is caused by skin atrophy and capillary fragility, whereas the prothrombotic state is due to an imbalance between the activity of pro- and anticoagulant pathways. In this overview the incidence, pathophysiology and prophylaxis of VTE associated with CS will be discussed.

## Incidence

Two phases are important with respect to the incidence of CS-associated VTE: the untreated active hypercortisolemic phase and the postoperative phase. In a systematic review, van Zaane and co-workers found an increased incidence rate of VTE in active CS (2.5–3.1 per 1000 person years) compared to the control population (0.27 per 1000 person years) [3]. In a systematic meta-analysis, VTE incidence in patients with CS was almost 18-fold higher compared to the general population [4], whereas a Swedish nation-wide study demonstrated a standardized incidence ratio for VTE of 13.8 (interquartile range 3.8–35.3) in the 3-year period before diagnosis of CS [5].

Postoperative VTE risk in the van Zaane study increased to 5.6%, comparable to the VTE risk after orthopedic surgery under routine thromboprophylaxis. Subsequently, a retrospective multicenter cohort study of 473 CS patients demonstrated an overall VTE incidence rate of 14.6 per 1000 person-years, which corresponds to more than a tenfold increased risk for VTE compared to the general population [6]. In this study the postoperative risk of VTE in patients with pituitary-dependent CS of 4.3% was increased compared to 0% incidence in patients operated for a nonfunctional pituitary adenoma [6]. Interestingly, no increased postoperative VTE risk was found in patients with ACTH-independent CS following adrenalectomy. In contrast, in a large retrospective study of 4217 patients who underwent adrenal surgery, Babic and co-workers found a VTE prevalence of 2.6% in patients (n = 310) with CS versus 0.9% in non-CS patients [7].

Interestingly, the postoperative VTE risk is not only increased in the first week after pituitary or adrenal surgery, but also up to 2–3 months or even years postoperatively, despite biochemical remission, which may indicate a delayed reversibility of the prothrombotic state [5–7].

### Pathogenesis

The hypercoagulable state in CS is not fully understood, but is thought to be caused by an imbalance between activity of pro- and anticoagulant pathways [2]. Indeed, functional assays show a shortening of the activated partial thromboplastin time (aPTT) and an increased clot lysis time in a subset of CS patients [2, 4]. In addition, concentrations of the procoagulant factors (fibrinogen, von Willebrand factor (vWF) and factor VIII) and fibrinolysis inhibitors (plasminogen activator inhibitor-1 (PAI-1), thrombin activatable fibrinolysis inhibitor (TAFI) and alpha-2-antiplasmin) [2, 4] are increased in active CS (Table 1.). Some studies show elevated levels of protein C, protein S and antithrombin III [4] which may be a compensatory mechanism for the hypercoagulable state. Glucocorticoids are thought to have direct stimulatory effects on the production of factors like fibrinogen, vWF, factor VIII, PAI-1 and TAFI [2], although no relation was found between severity of hypercortisolism and VTE risk in CS [4]. It should be emphasized, though, that reported coagulation profiles in CS patients are very heterogenous, presumably explained by individual patient characteristics and by differences in used assays that measure hemostatic parameters.

**Table 1:** Most consistent changes in functional hemostatic assays and plasma concentrations of pro-and anticoagulant factors in Cushing’s syndrome

Functional assays	
aPTT	↓
Clot lysis time	↑
Procoagulant factors	
Factor VIII	↑
Von Willebrand factor	↑
Fibrinogen	↑
Fibrinolysis inhibitors	
PAI-1	↑
TAFI	↑
α2-antiplasmin	↑
Anticoagulant factors	
Protein C	↑
Protein S	↑
Antithrombin III	↑

aPTT: activated partial thromboplastin time

PAI-1: plasminogen activator inhibitor-1

TAFI: thrombin activatable fibrinolysis inhibitor

Several studies show sustained abnormalities in pro- and anticoagulant factors after curative surgery in a subset of patients, which may in part explain the earlier mentioned prolonged VTE risk despite control of cortisol production. For instance, hemostatic parameters did not normalize after short-term biochemical remission, i.e. three months, induced by medical therapy [8]. Similarly, not all procoagulant factors normalized after longer follow-up, i.e. one year after surgical cure [9]. Persistent abdominal obesity, which is known to be associated with elevated plasma levels of fibrinogen, vWF and PAI-1, may underlie this prolonged elevation [10].

In addition to changes in hemostasis mediated by cortisol excess, other mechanisms may contribute to the increased VTE risk. After successful pituitary or adrenal surgery, a sudden fall in cortisol levels may lead to an inflammatory response [11] that can activate the coagulation cascade [12]. Further, in patients with ectopic ACTH production and patients with a cortisol producing adrenal carcinoma, the underlying malignant disorder itself predisposes for VTE. Retrospective studies have identified other more common risk factors for VTE, such as age, body mass index, reduced mobility, acute infections, previous cardiovascular events, midnight plasma cortisol level and a shortened aPTT [13].

### Thromboprophylaxis

To date, no prospective studies or guidelines have been published on efficacy, duration and type of thromboprophylaxis in active CS and in the postoperative period. However, retrospective studies have shown that the incidence of VTE was lower in patients who received thromboprophylaxis compared to those who did not [14, 15]. Apart from thromboprophylaxis, preoperative cortisol-lowering therapy may reduce VTE risk, although this was only shown in one retrospective study [6], whereas another retrospective study found no benefit [16]. A recent survey assessing current clinical practice of thromboprophylaxis in reference centers of the European Reference Network on Rare Endocrine Conditions (Endo-ERN) demonstrated considerable heterogeneity with respect to timing, duration and type of thromboprophylaxis [17]. Most centers started thrombophylaxis from diagnosis onwards, usually with low molecular weight heparin, but treatment duration was highly variable and often a thromboprophylaxis protocol was unavailable.

Currently the best approach may be to give thromboprophylaxis in a tailor-made manner in CS patients, both in the active phase and postoperatively, considering additional risk factors for thrombosis like history of VTE, age, obesity, current use of oestrogen or oral contraceptives and mobility, but also considering the risk of bleeding complications [18]. Further, it can be considered to temporarily stop oestrogen therapy in female patients before surgery. Thromboprophylaxis should be discontinued before transsphenoidal adenomectomy to minimize the risk of intraoperative bleeding [18]. In the postoperative setting, extended thromboprophylaxis, i.e. four to eight weeks, is recommended, especially for high-risk patients [2]. In addition, early postoperative ambulation and use of compression stockings are useful supportive measures to prevent VTE [18].

## Conclusion

CS is associated with an increased VTE risk. Currently there is no standard protocol for preoperative or postoperative thromboprophylaxis in patients with CS. Future studies should focus on the pathogenesis of the hypercoagulable state in CS and delineate which subgroups of CS are at greater risk for VTE based on clinical features and perhaps also based on profiles of coagulation parameters. This should be assessed for both the untreated active phase and the (extended) postoperative phase. Finally, there is a clear need for prospective studies that generate data that can guide the timing, duration and type of thromboprophylaxis in CS. The risk(s) for and the optimal prevention of life-threatening thrombotic events deserve more targeted investigation to improve morbidity and mortality.
